# Wavelength- or Polarization-Selective Thermal Infrared Detectors for Multi-Color or Polarimetric Imaging Using Plasmonics and Metamaterials

**DOI:** 10.3390/ma10050493

**Published:** 2017-05-04

**Authors:** Shinpei Ogawa, Masafumi Kimata

**Affiliations:** 1 Advanced Technology R&D Center, Mitsubishi Electric Corporation, Amagasaki 661-8661, Japan; 2 College of Science and Engineering, Ritsumeikan University, Kusatsu 525-8577, Japan; kimata@se.ritsumei.ac.jp

**Keywords:** thermal infrared, uncooled, infrared detector, wavelength-selective, polarization, plasmonics, metamaterials, absorber

## Abstract

Wavelength- or polarization-selective thermal infrared (IR) detectors are promising for various novel applications such as fire detection, gas analysis, multi-color imaging, multi-channel detectors, recognition of artificial objects in a natural environment, and facial recognition. However, these functions require additional filters or polarizers, which leads to high cost and technical difficulties related to integration of many different pixels in an array format. Plasmonic metamaterial absorbers (PMAs) can impart wavelength or polarization selectivity to conventional thermal IR detectors simply by controlling the surface geometry of the absorbers to produce surface plasmon resonances at designed wavelengths or polarizations. This enables integration of many different pixels in an array format without any filters or polarizers. We review our recent advances in wavelength- and polarization-selective thermal IR sensors using PMAs for multi-color or polarimetric imaging. The absorption mechanism defined by the surface structures is discussed for three types of PMAs—periodic crystals, metal-insulator-metal and mushroom-type PMAs—to demonstrate appropriate applications. Our wavelength- or polarization-selective uncooled IR sensors using various PMAs and multi-color image sensors are then described. Finally, high-performance mushroom-type PMAs are investigated. These advanced functional thermal IR detectors with wavelength or polarization selectivity will provide great benefits for a wide range of applications.

## 1. Introduction

Thermal infrared (IR) sensors are widely used for security, surveillance, search and rescue, firefighting, traffic systems, law enforcement, process control, and preventive maintenance [1,2,3]. These IR detectors are roughly divided into two categories: thermal and quantum (photon) detectors. Thermal detectors are widely used due to their low cost and simple operation without cooling.

Development is ongoing in industry in order to realize large-format [4], high-resolution [5] and low-cost applications [6]. Conventional thermal IR sensors detect averaged amounts of IR radiation from objects. Although light contains information such as wavelength, polarization and phase, thermal IR sensors utilize only intensity. Hence, there is rich potential to exploit wavelength and polarization information, which can elevate thermal IR detectors to the next stage and broaden their applications. Wavelength-selective thermal IR sensors can be used to identify objects through their spectral data, allowing for gas analysis, fire detection and eventually multi-color imaging [2]. Polarization-selective thermal IR sensors can enhance image recognition using polarimetric information, for instance, to improve object recognition and enable distinction between dissimilar objects or between artificial objects and the natural environment [7]. Thermal IR detectors with these advanced functions can not only detect but also identify and recognize thermal sources for what they are, which will provide great advantages in a wide range of fields.

Recently, advanced functional thermal IR detectors have drawn significant interest from both the field of applied physics and industry due to the emerging progress in nanophotonics subfields such as plasmonics [8,9,10,11], metamaterials [12,13,14], and plasmonic metamaterials [12,14]. To realize advanced functions, conventional technologies utilize the optical resonance structure between the absorber and the bottom reflector [15,16,17] or make use of filters and polarizers [18,19,20,21,22]. In contrast, advanced technologies realize novel functions for thermal IR sensors only by manipulating the surface geometry of the absorber, i.e., without attaching any filters and polarizers. Therefore, monolithic integration of pixels in an array format is possible.

In this paper, we review our recent results on multi-color or polarimetric thermal IR detectors using plasmonic and metamaterial absorbers. First, thermal IR sensors are briefly introduced. Absorbers are then discussed in order to realize advanced functions using plasmonics and metamaterials.

## 2. Thermal IR Detectors

First, we briefly explain the basics of thermal detectors.

**Figure 1:**
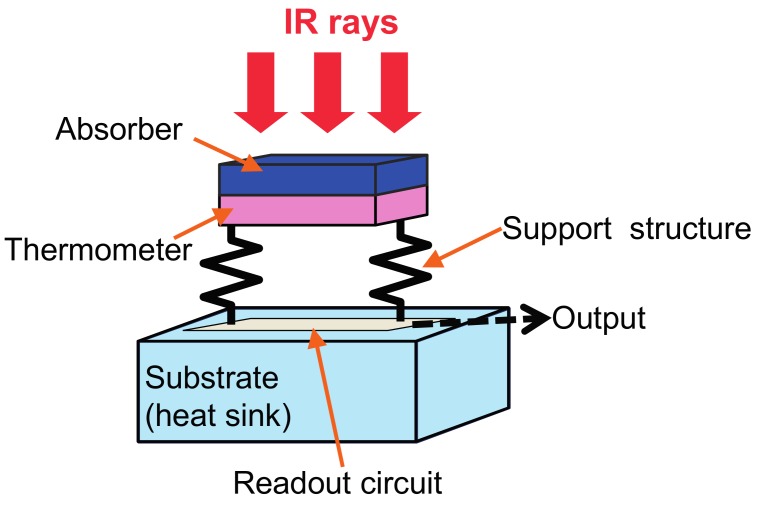
Operation principle of thermal IR detectors.

Figure 1 shows a schematic illustration of the general construction of a thermal IR detector, in which a thermometer is thermally and electrically coupled to a substrate on a support structure. This construction assumes an electrical readout. IR radiation is absorbed by an IR absorber attached to the thermometer, which changes its temperature. The support structure provides a mechanical support, thermal conducting paths to the substrate, and electrically conducting paths. If the thermal conductance of the support legs is sufficiently low, a detectable temperature change is generated at the thermometer. A change in the output from the thermometer is then read out through the support legs by the signal processing circuits on the substrate.

Thermal detectors are classified into six general groups based on their operating principles: (i) pyroelectric, based on temperature dependence of spontaneous polarization [23]; (ii) dielectric bolometer, based on temperature dependence of dielectric constant [24]; (iii) bolometer, based on temperature dependence of resistance [25,26,27]; (iv) thermoelectric, based on the Seebeck effect [28]; (v) diode, based on temperature dependence of I–V characteristics [4,29,30]; and (vi) mechanical displacement, based on bimaterial thermal stress [31,32,33]. The operating principle of the first five is the conversion of a temperature change into an electric signal. In the sixth, micro-cantilever structures composed of a bimaterial that bends due to the thermal stress of dissimilar materials [34] cause a capacitance change [31,32] or an optical reflectance change [33] by which the incident IR rays are detected.

It should be noted that wavelength- or polarization-selective absorbers can be applied for all of these thermal detectors. The type of thermal detector should be chosen according to the desired application and its cost.

## 3. Plasmonic Metamaterial Absorber

**Figure 2:**
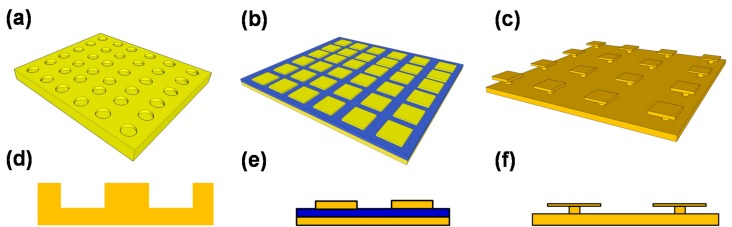
Schematic illustrations of the oblique and cross-sectional views of three types of PMAs: (**a**,**d**) Crystal-type; (**b**,**e**) MIM-type and (**c**,**f**) MR-type structures.

Recently, IR absorbers using plasmonics and metamaterials have been widely studied not only for uncooled IR sensors but also for IR emitters and radiative cooling. These absorbers are referred to by various names such as plasmonic absorbers, metamaterial absorbers, plasmonic metamaterial absorbers, metasurface absorbers or perfect absorbers [13,35]. Absorbers are reversely operated as emitters because of Kirchhoff’s law [13,36]. In this review paper, we limit the discussion to absorbers and call them plasmonic metamaterial absorbers (PMAs). PMAs are roughly classified into three types. Figure 2 shows oblique and cross-sectional views of these three types of PMAs: (a,d) crystal-type; (b,e) metal-insulator-metal (MIM)-type; and (c,f) mushroom-type (MR) structures. Each structure has advantages and disadvantages in terms of their optical properties and mass production.

Crystal-type absorbers are also called plasmonic crystals and have periodic dimpled structures, which are analogous to photonic crystals [37,38,39,40] rather than metamaterials. The periodic lattice structures define the absorption wavelength that controls the propagating surface plasmon resonance [41,42,43,44,45,46,47]. Such two-dimensional plasmonic absorbers (2D-PAs) have the advantages of simple fabrication and robustness of structural fluctuations, such as the dimple size and depth. The periodicity is crucially important in 2D-PAs [48]. The period can be completely maintained because it is defined only by photolithography. In one-dimensional (1D) or grating-PAs, the absorption wavelength is defined by the relative relationship between the width, the depth and the period [49,50,51,52,53]. Crystal-type PAs are suitable for single-pixel or small-format sensors for fire detection or gas sensing because of their incident angle dependence and large absorber volumes.

MIM-type PMAs (MIM-PMAs) are the most widely studied structures for a wide wavelength range covering the visible to the microwave [54,55,56,57,58,59,60,61,62,63,64,65,66,67,68,69,70,71,72,73,74,75,76,77,78,79,80,81,82,83,84,85,86,87]. MIM-PMAs basically consist of a bottom reflector layer, a middle insulator or semiconductor layer and a top-metal pattern. The total thickness of these layers is much thinner than the wavelength of light being detected. The magnetic response between these parallel plates produces strong localized surface plasmon resonance at the edges [88]. The absorption wavelength is defined mainly by the micropatch size and is larger than the period. MIM-PMAs are also called perfect absorbers because they are incident-angle and polarization insensitive due to the symmetric shape of the top metals. There are some variations of MIM-PMAs such as multi-layers [63,69,70,75] or multi-size micropatches [64,65,66,67] for broadband and multi-spectral absorption and complementary-perforated-surface structures [72,73,78,83]. MIM-PMAs are suitable for multi-spectral or polarimetric image sensors because of their small absorber area and volume. It should be noted that MIM-PMAs require highly precise control of the micropatch size to maintain the designed absorption wavelength [89].

Mushroom-type PMAs (MR-PMAs) consist of a bottom reflector and top isolated micropatches that are connected through posts [90,91,92,93,94], and were developed to advance MIM-PMAs. MIM-PMAs have additional undesired absorptions, non-linearity in the absorption wavelength and a gap wavelength region where wavelength-selective absorption cannot be induced due to the loss of insulator materials such as SiO_2_, Al_2_O_3_ or SiN [59,89,95]. In contrast, MR-PMAs have no middle insulator layer, which enables linear- and single-mode operation over a wide wavelength range, as well as a small absorber volume. Therefore, MR-PMAs are suitable for high-performance multi-color or polarimetric image sensors.

**Figure 3:**
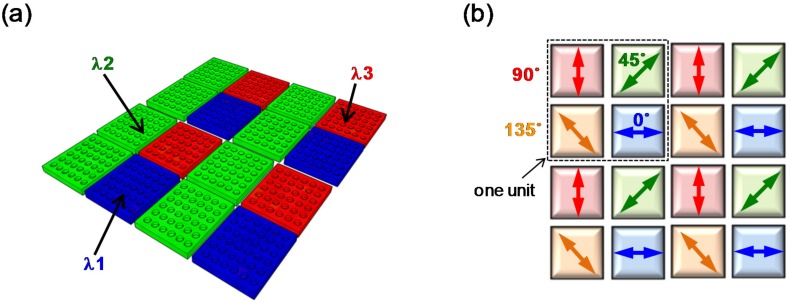
Concept of pixel integration in an array format for (**a**) multi-color; and (**b**) polarimetric imaging using PMAs.

Figure 3a,b show the concept of pixel integration in an array format with wavelength or polarization selectivity using PMAs for multi-color [42] and polarimetric imaging [44], respectively. Figure 3a shows the concept of PMAs with various surface pixel array structures, where three different PMAs with different absorption wavelengths (λ1, λ2, and λ3) are employed. Multi-color imaging can be realized by controlling only the surface structural parameters. Figure 3b shows the concept of the array structure for PMAs with polarization discrimination. One unit consists of four pixels and PMAs when different absorptions at polarization angle of 0°, 45°, 90°, and 135° are employed. Polarimetric imaging can thus be realized by introducing asymmetry to the surface structure.

## 4. Wavelength-Selective Function

**Figure 4:**
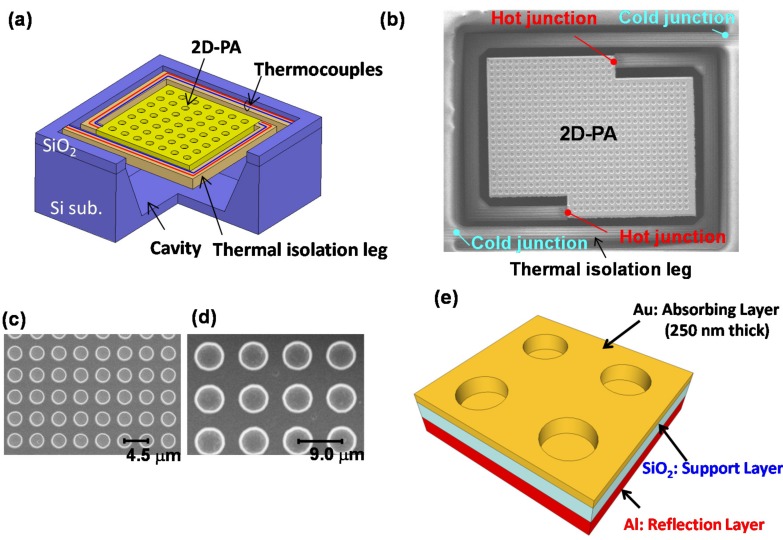
(**a**) Schematic diagram of the uncooled IR sensor (thermopile) with Au-based 2D-PA; (**b**) SEM image of the microelectromechanical systems (MEMS)-based thermopile with 2D-PA. Magnified SEM images of the 2D-PAs of (**c**) (ii) and (**d**) (vii); (**e**) Schematic diagram of the 2D-PA.

We have developed wavelength-selective uncooled IR sensors using Au-based 2D-PAs [41,42,43,45]. A thermopile was used for the uncooled IR sensor. Figure 4a,b show a schematic diagram and a scanning electron microscopy (SEM) image, respectively [41,42], of our developed wavelength-selective uncooled IR sensors using 2D-PAs with a square lattice. The fabrication procedure employed the conventional complimentary-metal-oxide-semiconductor (CMOS) process and a bulk micromachining technique. Various types of absorbers for the surface of the 2D-PAs can be chosen, such as Ag, Al, Au, Mo, and W plasmonic metals. Other plasmonic materials may be used according to the operating wavelength desired [96].

Various sensors with different 2D-PA structures were fabricated on the same wafer. The respective diameters and periods of the surface structures were as follows: (i) 3.0 and 4.0 μm; (ii) 3.0 and 4.5 μm; (iii) 4.0 and 5.0 μm; (iv) 4.0 and 6.5 μm; (v) 6.0 and 7.0 μm; (vi) 6.0 and 8.0 μm; (vii) 6.0 and 9.0 μm; and (viii) 6.0 and 10.5 μm. The depth was fixed at 1.5 μm for all sensors. Figure 4c,d show the SEM images of (ii) and (vii), respectively [42]. As shown in Figure 4e, an Al layer was formed at the bottom of the 2D-PA, which prevents the back side SiO_2_ layer from absorbing scattered light in the long-wavelength IR (LWIR) region [42]. The absorption by the material itself used in a sensor, except the absorber surface, produces absorption at additional wavelengths and polarization-insensitive absorption, which degrade the wavelength or polarization selectivity.

**Figure 5:**
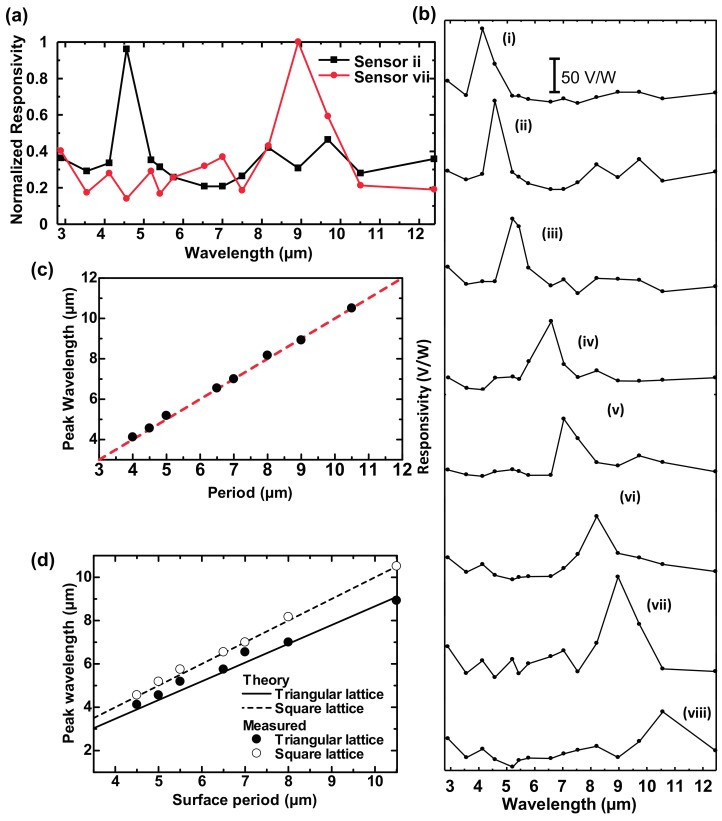
Measured spectral responsivity of developed sensors (**a**) (ii) and (vii); and (**b**) (i) to (viii); (**c**) Peak wavelength of the responsivity as a function of surface period for 2D-PA with square lattice; (**d**) Comparison of peak responsivity vs. surface period for 2D-PA square and triangular lattices.

Figure 5a,b show the measured spectral responsivity of sensors (ii) and (vii), and of all sensors (i)–(viii), respectively [42]. These results clearly show that wavelength selectivity was achieved for all sensors simply by controlling the surface geometry with the same dimple depth. Figure 5c shows the relationship between the peak responsivity and the surface period of 2D-PAs with a square lattice [42]. The measured results clearly demonstrate that wavelength selectivity was achieved for a wide wavelength range covering the middle-wavelength IR (MWIR) and LWIR regions. Figure 5d compares the theoretical and experimental peak wavelength vs. surface period results for 2D-PAs with the square and triangular lattices [45]. These results indicate that the peak absorption wavelength can be defined by the reciprocal lattice vector of 2D periodic structures.

**Figure 6:**
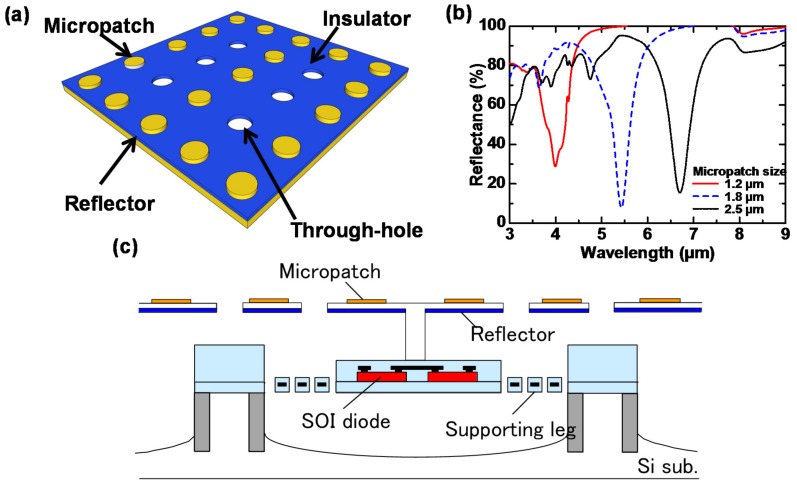
(**a**) Schematic of TH MIM-PMAs; (**b**) Measured reflectance of MIM-PMAs with various micropatch sizes; (**c**) Pixel structure of SOI diode uncooled IRFPA with TH MIM-PMAs.

Next, we applied MIM-PMAs in order to realize image sensors [76]. We developed a SOI diode uncooled IR focal plane array (IRFPA) using through-hole (TH) MIM-PMAs. Through-holes can be integrated in MIM structures while maintaining wavelength selectivity due to their antenna effect. Figure 6a,b show a schematic diagram of one TH MIM-PMA and the reflectance of MIM-PMAs for various micropatch sizes, respectively [76]. The reflectance dip corresponds directly to the absorption because the reflector of a MIM-PMA is thick enough to prevent transmission, so the absorbance is obtained as (1—reflectance). Figure 6b shows that the absorption wavelength can be controlled by the micropatch size to a lesser extent than by the period. Figure 6c shows the pixel structure of the SOI diode uncooled IRFPA with TH MIM-PMAs, which was fabricated with a fully dry bulk/surface combined micromachining process using organic sacrificial layers [76].

**Figure 7:**
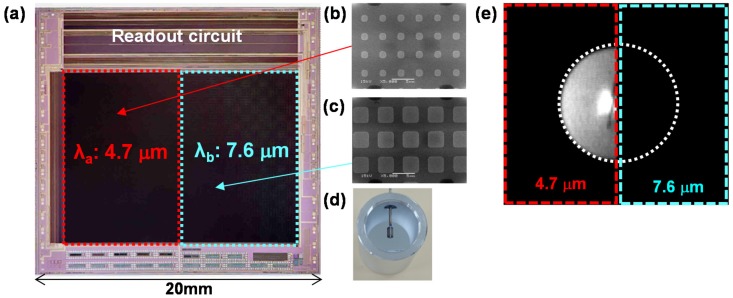
(**a**) Optical microscopy image of the developed image sensor with two TH MIM-PMAs. SEM images of MIM-PMAs integrated in the (**b**) left and (**c**) right halves of the pixel array; (**d**) Optical image of the light emitter used in the image; (**e**) Image of the light emitter obtained using the developed image sensor with the narrow bandpass filter centered at 4.7 μm.

Two-color image sensors were then fabricated [97]. Figure 7a shows an optical image of the fabricated 50 μm-pixel-pitch 320 × 240 SOI diode IRFPA with TH MIM-PMAs, which has dimensions of 20.0 × 19.0 mm^2^. Figure 7b,c show SEM images of MIM-PMAs for the left and right halves of the 320 × 240 pixel array, which detect wavelengths of 4.7 and 7.6 μm, respectively. Figure 7e shows the image of the light emitter as shown in Figure 7d taken by the developed sensor. The light emitter was attached with a narrow bandpass filter with a center wavelength of 4.7 μm, which corresponds to the absorption wavelength of the left half of the pixel array.

Figure 7 clearly demonstrates that our developed multi-color image sensor can take spectral images according to the absorption wavelength of the MIM-PMAs. More pixels with different detection wavelengths can be integrated into the pixel array simply by controlling the surface pattern of MIM-PMAs through conventional photolithography.

## 5. Polarization-Selective Function

Polarization selectivity can be achieved using PMAs if a symmetry-breaking structure is introduced to the two orthogonal directions in the absorber surface plane. Possible examples include: (1) changing the dimple shape from a circle to an elliptical shape; (2) changing the dimension of the dimple periodicity from 2D to 1D for 2D-PAs; (3) changing the micropatch shape from a circle or square to a rectangle or ellipse; and (4) changing the dimension of the micropatch periodicity from 2D to 1D for MIM-PMAs and MR-PMAs. This geometric asymmetry in the absorber surface plane produces asymmetric behavior for the incident IR rays, which enables polarization-selective detection.

**Figure 8:**
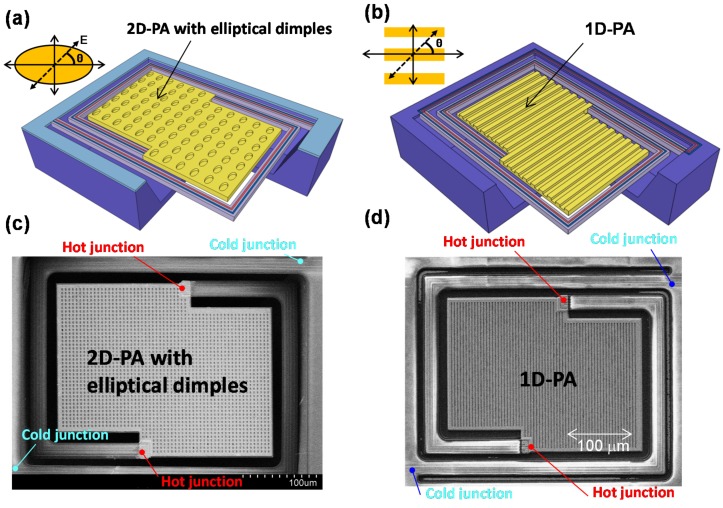
Schematic and SEM images of the uncooled IR sensor (thermopile) using (**a**,**c**) Au-based 2D-PA with ellipsoidal dimples; and (**b**,**d**) 1D-PA. The definition of the electric field polarization angle (θ) was defined according to the axis of the ellipsoid and the gratings.

Figure 8a–d show schematic and SEM images of our developed polarization-selective uncooled IR sensors with 2D-PAs incorporating elliptical dimples [44] and 1D-PAs [52], respectively. The polarization angle of the incident electric field was defined as θ.

**Figure 9:**
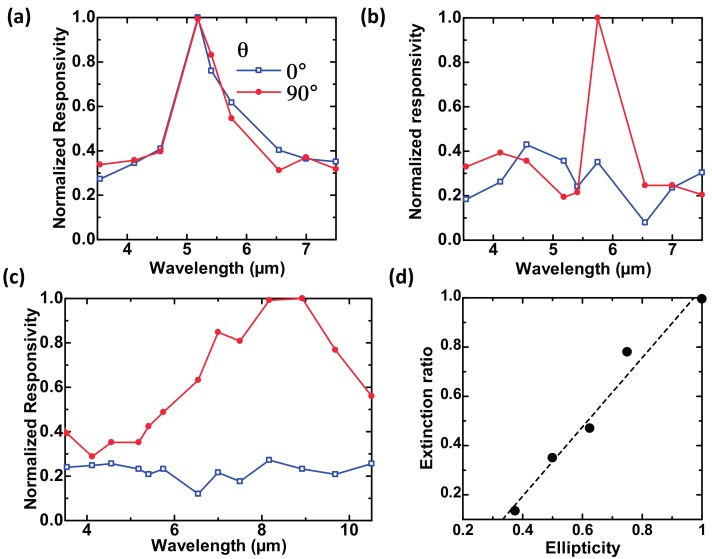
Measured polarization dependence of spectral responsivity of developed uncooled IR sensors using 2D-PAs with (**a**) circular and (**b**) ellipsoidal dimples, and with (**c**) 1D-PAs. (**d**) Relationship between the extinction ratio and the ellipticity.

The polarization dependence of spectral responsivity was measured for these sensors. Figure 9a–c show the measured results for 2D-PAs with circular (symmetric) and elliptical (asymmetric) dimples [44] and 1D-PAs [52], respectively. The diameter of the circular dimples was 4.0 μm. The lengths of the major and the minor axes were fixed at 4.0 and 2.0 μm for the elliptical dimples, respectively. The width of the grating grooves was 3.0 μm for the 1D-PA. The period and depth were fixed at 5.0 and 1.5 μm for all sensors.

Figure 9a–c demonstrate that polarization selectivity was realized as a result of the surface asymmetry. 2D-PAs have two periods in the absorber surface plane; the period in the plane direction more strongly influences the plasmonic resonance than that in the dimple depth direction. The polarization-selective absorption wavelength can be defined by the period [41,42]. However, 1D-PAs have more influence on the plasmonic resonance in the depth direction with mixing than in the surface direction, which produces broader polarization selectivity [52].

The extinction ratio was defined as the ratio of responsivity of the electric field at θ = 0° to that at θ = 90° at the wavelength of maximum responsivity. Figure 9d shows the relationship between the extinction ratio and the ellipticity [44]. The ellipticity was defined as the ratio of the minor axis length to the major axis length of the ellipsoid. These results indicate that strong asymmetry produces better polarization selectivity.

The same concept can be applied to MIM and MR-PMAs with rectangular or elliptical micropatches in 2D structures or striped micropatches in 1D structures.

## 6. High-Performance Absorbers

It is critical to select appropriate PMAs for thermal IR detectors considering the field of application, number of pixels, cost and type of integrated system. MIM-PMAs are considered to be high-performance absorbers due to their thin, small and incident angle insensitive properties. However, the loss of the insulator material degrades the wavelength or polarization selectivity and causes additional polarization-insensitive absorption. Lossless materials such as ZnS [66] or CeO_2_ [87] are not compatible with the CMOS process and cause additional polarization-insensitive peaks due to the waveguide mode in the insulator layer. Therefore, we investigated MR-PMAs to address this challenge [90,91,92,93].

**Figure 10:**
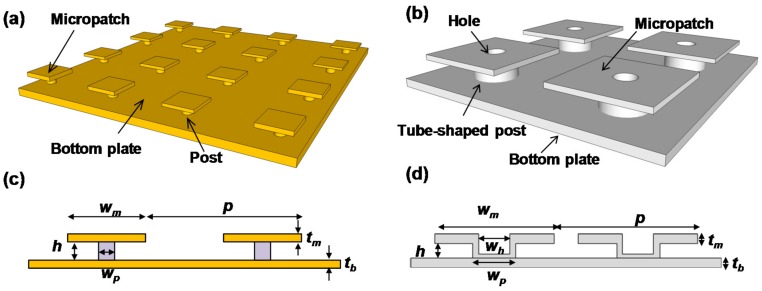
Schematic and cross-sectional images of MR-PMAs with (**a**,**c**) cylindrical and (**b**,**d**) tubular posts.

Figure 10a–d show schematic and cross-sectional diagrams of MR-PMAs with cylindrical [92] and tubular posts [93], respectively. The period, width and thickness of the micropatches, the inner diameter of the tube-shaped metal posts, width and height of the posts, and the thickness of the bottom plate are denoted as *p*, *w_m_*, *t_m_*, *w_h_*, *w_p_*, *h* and *t_b_*, respectively. Here, *w_p_* is defined as the sum of *w_h_* and the Al sidewall thickness of 100 nm, based on a consideration of the fabrication method.

The absorbance of these structures was calculated using the rigorous-coupled wave analysis (RCWA) method.

**Figure 11:**
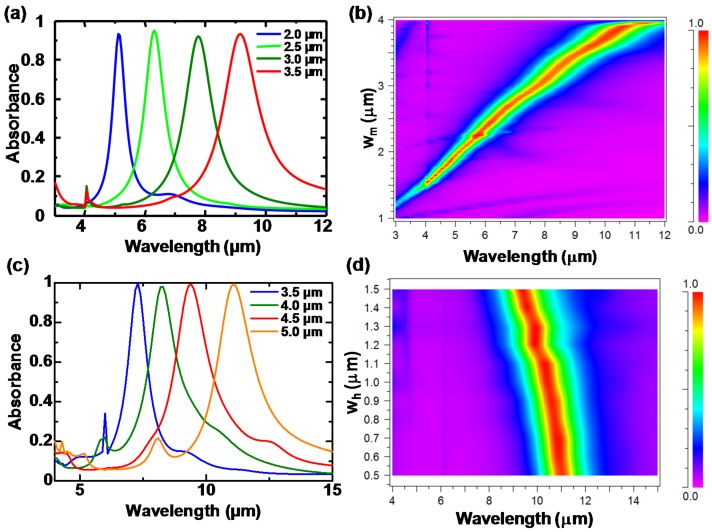
Calculated absorbance spectra of MR-PMAs with cylindrical posts. (**a**) Spectra for various *w_m_* and (**b**) as a function of wavelength and *w_m_*. Calculated absorbance of MR-PMAs with tubular posts; (**c**) Spectra for various *w_m_* and (**d**) as a function of wavelength and *w_h_*. The color map defines the absorbance scale.

Figure 11a,b show the calculated absorbance spectra of all-metal Au-based MR-PMAs with cylindrical posts for various *w_m_*, and as a function of the wavelength and *w_m_*, with fixed *p*, *t_m_*, *w_p_*, *h* and *t_b_* of 4.0 μm, 50 nm, 200 nm, 150 nm and 100 nm, respectively [92]. Figure 11a,b demonstrate that MR-PMAs with cylindrical posts realize nearly linear wavelength-selective absorption without additional absorption peaks. However, the fabrication of these structures is complicated and requires many steps due to the necessity of the multi-step sacrificial etching method [90]. We proposed two types of post structures to address this issue: Si posts [91] and tubular posts [93]. MR-PMAs with Si posts can be simply fabricated by XeF_2_ etching of the Si insulator layer in MIM structures. The MR-PMAs with Si posts can suppress the resonant mode in the posts by reducing *w_p_*, which can be varied to tune the single- or the dual-band detections.

MR-PMAs with tubular posts can be fabricated using only one sacrificial etching. Figure 11c,d show the calculated absorbance spectra of all-metal Al-based MR-PMAs with tubular posts for various *w_m_*, with fixed *p*, *t_m_*, *w_h_*, *h* and *t_b_* of 6.0 µm, 100 nm, 1.3 µm, 200 nm, and 100 nm, and as a function of wavelength and *w_h_* for fixed *w_m_* = 4.5 µm, respectively [93]. These results demonstrate that strong wavelength-selective absorption can be realized in the LWIR region and causes no additional absorption regardless of the tubular post structures. Furthermore, the absorption wavelength can be tuned by *w_m_* and *w_h_*.

**Figure 12:**
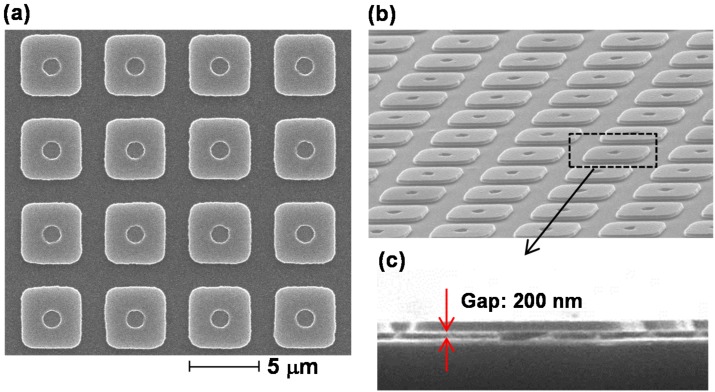
SEM images of MR-PMAs with tubular posts: (**a**) top; (**b**) oblique; and (**c**) magnified cross-sectional views of the periodic structures.

Figure 12a–c show SEM images of the developed MR-PMAs with the tubular posts [93]. All of our micropatches were successfully fabricated without bending or sticking. These images clearly demonstrate that a uniform gap size of 200 nm was achieved between the micropatches and the bottom plate despite the presence of the tubular post structures, providing strong plasmonic resonance. This fabrication procedure is also compatible with CMOS technology.

**Figure 13:**
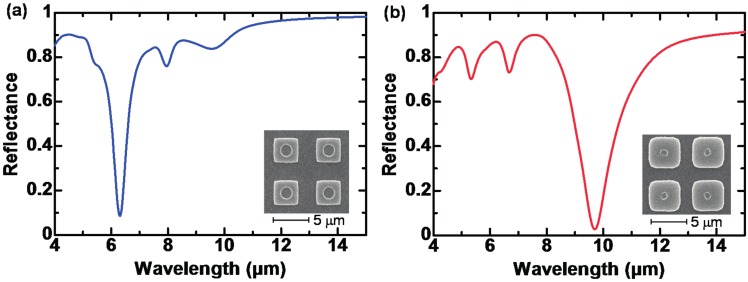
Experimental reflectance spectra for devices with *w_m_* and *w_h_* of (**a**) 3.5 and 1.5 µm; and (**b**) 4.3 and 0.7 µm.

Figure 13a,b show the measured reflectance of the developed MR-PMAs with *w_m_* and *w_h_* of 3.5 and 1.5 µm, and 4.3 and 0.7 µm, respectively, with fixed *p* = 6.0 µm [93]. The reflectance dip directly corresponds to the absorption peaks, as in the case of MIM-PMAs.

Figure 13a,b demonstrate that the wavelength-selective absorption can be controlled by *w_m_* and *w_h_*, and that strong absorption is obtained at 9.7 µm with a very high absorbance of over 95%. Due to the tolerance in the period and the thin absorber thickness, a small pixel size can be achieved, which leads to high resolution and a fast response. The MR-PMAs are good candidates for high-performance wavelength- or polarization-selective absorbers due to the wide operating ranges in the MWIR and LWIR without disturbance of the non-linear effect of the insulator loss.

## 7. Conclusions

We reviewed the recent advances of our wavelength- and polarization-selective thermal IR detectors using plasmonics and metamaterials technologies. The basics of thermal IR detectors were introduced. Absorbers using plasmonics and metamaterials were classified into three categories and discussed in terms of their structures, advantages and appropriate applications. Our developed wavelength-selective uncooled IR sensors, multi-color IR image sensors, and polarization-selective uncooled IR sensors were described. Additionally, mushroom absorbers were discussed for high-performance multi-color or polarimetric imaging. These advanced functional thermal IR detectors with wavelength or polarization selectivity will contribute to expand IR sensor applications and provide great benefits to our society over a wide range of applications, such as fire detection, gas analysis, material identification, and object recognition. We also believe that the basic principles are applicable for the terahertz region.
